# Microbiome Changes in Layer Pullets Reared in Floor Pens along the Growth Period

**DOI:** 10.3390/life13122302

**Published:** 2023-12-05

**Authors:** Hee-Jin Kim, Hyun-Soo Kim, Yeon-Seo Yun, Hyekyoung Shin, Woo-Do Lee, Jiseon Son, Eui-Chul Hong, Ik-Soo Jeon, Hwan-Ku Kang

**Affiliations:** Poultry Research Institute, National Institute of Animal Science, Rural Development Administration, Pyeongchang 25342, Republic of Korea; khj0175@korea.kr (H.-J.K.); kindkims1@korea.kr (H.-S.K.); yys1313@korea.kr (Y.-S.Y.); woodo92@korea.kr (W.-D.L.); jeonis@korea.kr (I.-S.J.)

**Keywords:** gut microbiome, pullet, floor, layer hen, rearing period

## Abstract

The gastrointestinal tract microbiome is essential for regulating nutrient absorption, gut immune function, and host growth and development. In the present study, we characterized the development of ileum and cecum microbiota in pullets throughout the rearing period, encompassing a period from the day of hatching to 18 weeks of age. The growth performance and intestinal microbiome (ileum and cecum) of pullets were analyzed at 1, 5, 11, and 18 weeks of age. The richness of the ileum and cecum bacterial communities (alpha diversity) was higher in pullets at 18 weeks of age than in those at 1 and 5 weeks of age. Microbiota from weeks 1, 5, 11, and 18 were distinctly grouped in a NMDS plot, representing beta diversity within the ileum. However, the results for cecum microbiota did not reveal evident separation among the different age groups in the weighted UniFrac. In conclusion, our findings demonstrate variations and diversification in ileum and cecum microbiota across different rearing stages in pullets. These insights have the potential to inform the development of nutritional strategies that promote gut health and contribute to the improved development of pullets.

## 1. Introduction

Laying hens provide humans with the primary protein source through their eggs. The use of antibiotic growth promoters has recently been banned, leading to a surge in research efforts to discover viable alternatives that can effectively promote the growth of chickens. As an alternative, additional advantageous and commonly used supplements for laying hens encompass amino acids, vitamins, minerals (such as calcium and zinc), prebiotics (primarily fiber-based), probiotics, feed enzymes (like xylanase and β-glucanase), and antioxidants [1]. In particular, an increasing amount of research has been conducted on additives associated with gut microbiota and their influence on gut health in chickens. Maintaining healthy intestinal conditions in layer hens before they start laying eggs is essential and beneficial as it creates an environment for improved laying performance throughout their lifespan [2]. The small intestine (ileum) serves as a crucial organ essential for sustaining digestive, endocrine, metabolic, and immune functions in domestic animals. Within this context, the intestinal physical barrier, composed of intestinal epithelial cells and junctional complexes, assumes a pivotal role in both nutrient absorption and safeguarding the gut from potentially harmful substances, including endotoxins and pathogens [3]. Also, the intricate nature of ileum microbiota holds significance in the preservation of gut health, its direct influence on the process of digestion, and its capacity to shape the overall production performance of chickens [4]. The microorganisms present in the cecum have the capacity to metabolize undigested nutrients into beneficial end products. These end products play vital roles in promoting gut development, supporting the maturation of the immune system, and facilitating the absorption of essential nutrients [5].

Resident microbiota in the gastrointestinal (GIT) tract can affect host health and susceptibility to diseases through the interplay of metabolic dynamics and immune-inflammatory pathways [6,7]. The GIT of broilers hosts an impressive array of over 900 bacterial species, each playing a vital role in various processes such as food digestion, detoxification, immune system support, pathogen prevention, and hormonal regulation [8]. Microbiota and bacterial fermentation in the GIT play vital roles in enhancing nutrient absorption, neutralizing toxins, and preventing the establishment of harmful pathogens [8]. These factors are essential to bolstering pathogen resistance and regulating the overall health and performance of chickens [9]. Moreover, gut microbiota offer health benefits by restraining the occurrence of chronic ailments [10], protecting the host against a range of pathogens [11], and promoting optimal gut well-being [12]. Additionally, gut microbiota are essential in expelling pathogens from the host and facilitating the development of the host immune system [6,13]. Also, as feed is ingested and passes through the GIT, various microbial groups come into play, initiating the crucial process of digestion [14]. The GIT of chickens can be segmented into three parts: the upper segment, small intestine, and large intestine, all of which are colonized by microbial populations throughout their entire length [14]. While it is common to study each section as an independent ecosystem due to the significant diversity of those sections, it is important to acknowledge that these segments are intricately interconnected and can mutually influence the composition of one another’s microbial communities [14]. Microbial communities within the gastrointestinal tract have evolved alongside their host organisms to an extent, and it has been proposed that the composition of an individual’s gut microbiota can be as distinct and unique as their fingerprint is [15].

Additionally, the growing trend toward cage-free environments for egg-laying chickens necessitates innovative approaches to maintain their health and prevent diseases [2]. A substantial portion of the GIT microbiota remains relatively unknown, presenting a significant reservoir of untapped biological potential. This includes the discovery of previously unknown bacterial species, their encoded enzymatic activities, and the possibility of identifying probiotic bacterial strains. Therefore, gaining a deeper understanding of the composition and dynamic distribution of ileum and cecum microbiota in hens raised on the floor, in conjunction with their growth and development, could offer new insights into gut development and healthcare. Previous studies have also indicated that comprehensive analyses of the gut microbiota at various developmental stages can enhance our understanding of the effective selection and use of probiotics during specific physiological phases [2,16,17,18]. Despite the advancements, there remains a considerable knowledge gap concerning egg-laying birds, notably those in the pullet phase raised on the floor. Moreover, there is limited research dedicated to exploring the gut microbiota in the ileum and cecum, specifically regarding variations associated with age within these gut microbiota. In our current study, we sought to address this knowledge gap by investigating the developmental changes in the ileum and cecum microbiomes of pullets.

The current study was performed to comprehensively define the ileum and cecum bacterial microbiota in Hy-Line Brown pullets maintained on the floor under a standard management protocol. This study aimed to offer new perspectives for the creation and enhancement of nutritional strategies for the effective management of intestinal health and development in laying pullets. Furthermore, to address the dynamic shifts in physiological traits and nutritional demands of pullets, we subdivided the pre-laying phase (0–18 weeks) into distinct periods that aligned with critical aspects of their development. Specifically, we focused on digestion and immune system maturation during the initial 0–5 weeks, bone development from 6 to 11 weeks, and the critical stages of genital and reproductive system development in the subsequent 12–18 weeks.

## 2. Materials and Methods

### 2.1. Animals and Housing Conditions

The animal experimental protocol was reviewed and approved by the Institutional Animal Care and Welfare Committee of the National Institute of Animal Science, Rural Development Administration, Republic of Korea (Approval Number 2021-508). The eggs from the Hy-Line Brown strain were sourced from a well-established hatching company. Approximately 516 chicks were raised under conventional pullet rearing conditions in floor pens with unrestricted access to both water and commercial feed. These birds were weighed individually and randomly assigned to floor pens at a rate of 86 birds/pen. Each floor was replicated 6 times. During the initial growth period from 0 to 5 weeks, the pullets were supplied with a starter diet containing 2850 kcal/kg of metabolizable energy, 18.5% crude protein, and 1.1% calcium. Subsequently, from weeks 6 to 12, their diet transitioned to a grower diet with 2820 kcal/kg of metabolizable energy, 16.5% crude protein, and 1.0% calcium. As they approached the pre-laying stage, spanning weeks 13 to 18, they were provided with a pre-lay diet featuring 2800 kcal/kg of metabolizable energy, 15.0% crude protein, and 1.8% calcium. The feed did not contain antibiotics, coccidiostats, or growth hormones. During the initial week, the room’s temperature was maintained at 34–36 °C. Subsequently, the temperature was gradually reduced by 2 °C each week until it reached approximately 22 °C. Body weight was measured every two weeks. The lighting schedule commenced with an initial intensity of 50 lux (22 h of light:2 h of darkness). The lighting regimen was tailored following the management recommendations for Hy-Line Brown laying hens from week 0 to week 18.

### 2.2. Cecum and Ileum Sampling

At the specified ages of 1, 5, 11, and 18 weeks, we conducted humane euthanasia on five birds with approximately average body weights by employing CO_2_. Euthanasia was followed by placing each bird in the supine position, and with the utmost care, the body cavity was surgically accessed using sterilized alcohol-flamed scissors. Immediately thereafter, the freshly obtained cecum and ileum contents were collected and promptly frozen via immersion in liquid nitrogen. These samples were subsequently preserved at a temperature of −80 °C to ensure their preservation for future analyses.

### 2.3. DNA Extraction and Microbiome 16S rRNA Sequencing

Microbiota genomic DNA was extracted from the cecum and ileum contents using POWERSoil DNA Kit (QIAGEN, Hilden, Germany) in accordance with the manufacturer’s instructions. The evaluation of the extracted DNA’s overall quality, which included the assessment of base sequence quality, GC content, quality score, and N content, was carried out using FastQC (v0.10.1) software. The V3–V4 hypervariable regions of the 16S rRNA gene were amplified using fusion primers.

### 2.4. Microbiome Diversity and Abundance Analyses

For the alpha microbial community, ANOVA analysis was performed for multiple mean comparisons between spawning stages, followed by Tukey’s test. A significance level of *p* value ≤ 0.05 was considered statistically significant. For better diversity, Bray–Curtis metrics were utilized, representing quantitative and qualitative dissimilarity measures. Additionally, non-metric multidimensional scaling (NMDS) plots were generated based on (weighted/unweighted) UniFrac distance metrics considering the phylogenetic relationships. To ascertain group differences, permutation multivariate analysis of variance (PERMANOVA) was conducted as part of the statistical analysis. To identify bacteria exhibiting significant differences between the comparison groups, the composition of the microbiomes was analyzed using the bias correction (ANCOM-BC) statistical method.

### 2.5. LEfSe Analysis

The linear discriminant analysis (LDA) of effect size (LEfSe) (http://huttenhower.sph.harvard.edu/LEfSe: accessed on 4 November 2023) was performed to identify the significantly abundant taxa (phylum to genus) of bacteria among the different groups (LDA score > 2, *p* ≤ 0.05) as described previously by Segata et al. [19]. LDA uses log-transformed LDA scores with a threshold of 2.0 to separate samples and identifies the effect size of these groups in differentiating samples. A LEfSe score exceeding 2.0 was deemed significant.

## 3. Results

### 3.1. Weight

**Figure 1:**
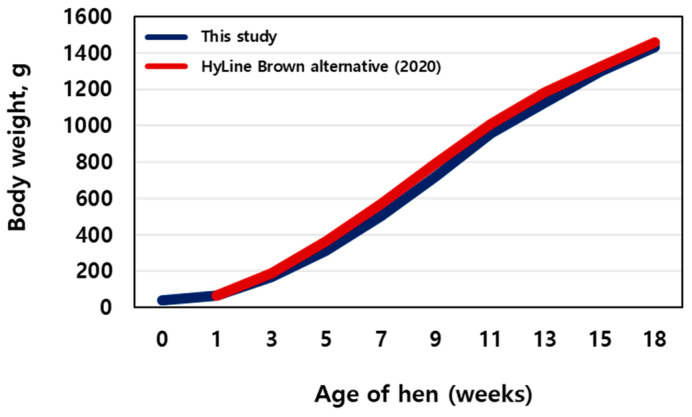
Average body weights (of sampled birds) during rearing (n = 400).

As shown in Figure 1, with advancing age in the pullets, body weight increased (blue line). The chicken body weights at different growth stages were 67.90 ± 1.47, 313.01 ± 32.40, 860.48 ± 103.86, and 1499.63 ± 101.93 g at 1, 5, 11, and 18 days, respectively. A similar trend was observed for the Hy-Line Brown alternatives (red line). No mortality was observed during the trial.

### 3.2. Alpha Diversity

As shown in Table 1, the alpha-diversity index of the ileum microbiome was summarized based on Chao1, Shannon entropy, Simpson, and Faith pd. Higher species richness values (Chao1 and Faith pd) were determined in pullets at 18 weeks of age than it was in those at 1 and 5 weeks of age (*p* ≤ 0.05), and bacterial diversity (Shannon entropy and Simpson) was not significantly different among the ages (*p* > 0.05) in the ileum.

As shown in Table 2, the alpha-diversity index of the cecum microbiome was summarized based on Chao1, Shannon entropy, Simpson, and Faith pd. Higher species richness values (Chao1 and Faith pd) were observed in pullets at 11 and 18 weeks of age than in those at 1 and 5 weeks of age (*p* ≤ 0.05), and bacterial diversity (Shannon entropy and Simpson) exhibited a similar phenomenon. These findings suggest that as pullets grow and develop, both the diversity and abundance of cecum microbes increase.

### 3.3. Beta Diversity

**Figure 2:**
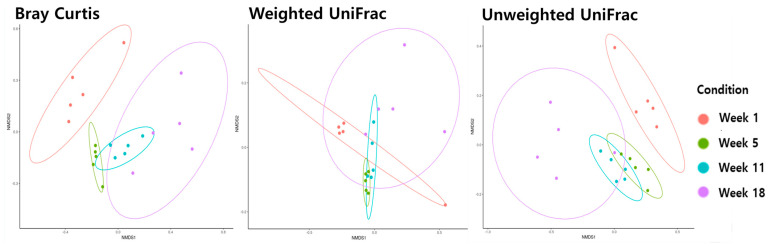
The beta diversity of the ileum microbiome during different stages of pullets for the 18 weeks.

**Figure 3:**
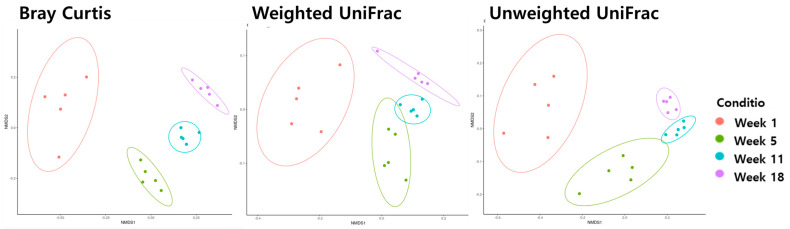
The beta diversity of the cecum microbiome during different stages of pullets for the first 18 weeks.

Beta diversity analysis using Bray−Curtis, weighted UniFrac, and unweighted UniFrac metrics was performed to analyze the distance between the ages of the microbiota in the ileum (Figure 2) and cecum (Figure 3). Age was significantly correlated with changes in microbiota composition (PER-MANOVA using Bray−Curtis, weighted, and unweighted UniFrac metrics; all at *p* ≤ 0.05). We utilized a principal coordinate analysis based on Bray−Curtis distances to assess al-terations in community structure over different ages of microbiota in the ileum. The results of the weighted UniFrac similarity indices were evidenced by similar cluster formation in different groups in the cecum. However, the results showed an evident separation between the ileum of the age groups in the unweighted UniFrac indices.

The microbiota at weeks 1, 5, 11, and 18 were grouped separately on all NMDS plots (Bray−Curtis, weighted UniFrac, and unweighted UniFrac; all *p* ≤ 0.05) in the cecum.

### 3.4. Composition of Gut Bacteria

**Figure 4:**
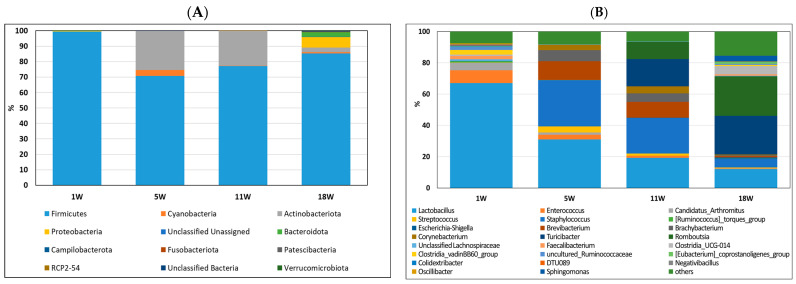
Relative abundance of bacterial composition in ileum contents at phylum (**A**) and genus (**B**) levels among different stages of pullets at 1, 5, 11, and 18 weeks. Each color represents one bacterium. The *x*-axis shows the age of pullets in weeks, and the *y*-axis shows the percentage of bacteria.

**Figure 5:**
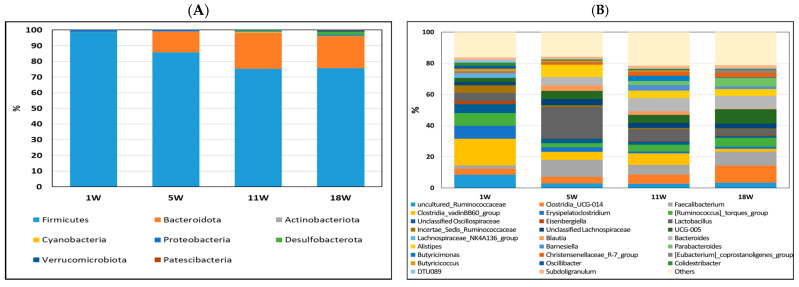
Relative abundance of bacterial composition in cecum contents at phylum (**A**) and genus (**B**) levels among different stages of pullets at 1, 5, 11, and 18 weeks. Each color represents one bacterium. The *x*−axis shows age of pullets in weeks, and the *y*−axis shows the percentage of bacteria.

To investigate the cecum microbiota composition of pullets at different developmental stages before egg laying, the composition and abundance of the ileum (Figure 4) and cecum microbiota (Figure 5) were compared at 1, 5, 11, and 18 weeks of age. As shown in Figure 4A, the most abundant phylum in the ileum at 1 week was *Firmicutes* (99.60%). However, the most abundant phyla in the ileum contents at 5 and 11 weeks were Firmicutes (70.81−77.18%) and *Actinobacteriota* (22.51−25.81%). More diverse microbiota, including *Cyanobacteria* (0.92%), *Bacteroidetes* (3.03%), and *Proteobacteria* (6.66%), were found in the ileum microbiota of 18-week-old pullets. At the genus level, *Lactobacillus* (67.01%), *Enterococcus* (8.17%), and *Candidatus Arthromitus* (4.91%) were the major bacterial genera in pullets at one week of age (Figure 4B). However, *Lactobacillus*, *Staphylococcus*, *Turicibacter*, and *Romboutsia* were the major bacterial genera in the ileum contents at 11 and 18 weeks of age.

The most abundant phyla in all periods were *Firmicutes* (75.58−98.60%) and *Bacteroidetes* (0−22.87%) (Figure 5A). However, the proportions of each of these phyla among the four periods differed. The percentage of *Firmicutes* increased, and that of *Bacteroidetes* decreased in the first 5 weeks and then remained stable from 11 to 18 weeks. During the fifth week, a noteworthy shift was observed in the cecum microbiota, with the relative abundance of *Bacteroidota* increasing from 0% to 13.3%. Simultaneously, within the ileum, there was an increase in the relative abundance of *Actinobacteriota* from 0% to 25.3%. As the third most abundant phylum, *Desulfobacterota* significantly increased to 2% of the total cecum microbes in the 18-week-old pullets. At 1 week of age, the *Clostridia* vadinBB60 group (17.08%) was the most major bacterium; at 5 weeks of age, *Lactobacillus* (20.02%) was the most major bacterium; at 11 weeks of age, *Bacteroides* (8.49%) was the most major bacterium, and at 8 weeks of age, *Clostridia* UCG-014 (11.10%) was the major bacterium. Based on the relative abundance results, a significant shift in microbiota was observed in pullets at 1, 5, 11, and 18 weeks of age.

### 3.5. LDA Analysis

**Figure 6:**
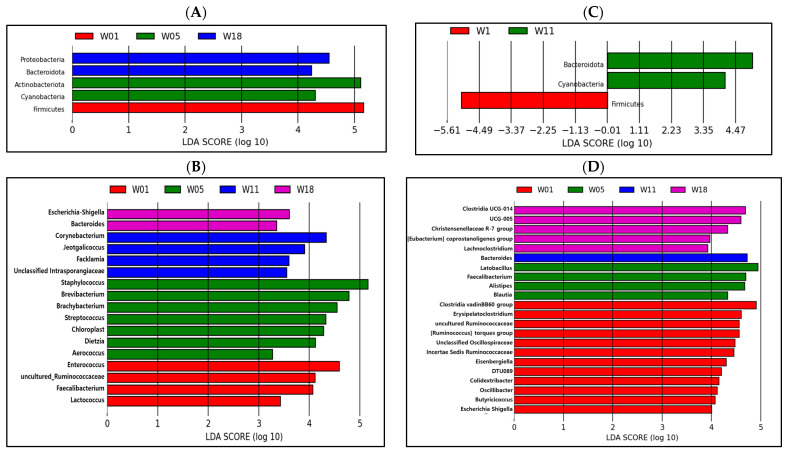
Microbe analysis of different taxa in the ileum ((**A**) phylum; (**B**) genus) and cecum ((**C**) phylum; (**D**) genus) based on the LEfSe method in pullets at 1, 5, 11, and 18 weeks. The default parameters were LDA score >2 and *p* ≤ 0.05. Bacteria with red, green, blue, or purple colors are higher at 1, 5, 11, and 18 weeks, respectively.

From the LEfSe analysis shown in Figure 6A, a higher abundance of *Firmicutes* was found at 1 week, a higher abundance of *Actinobacteria* and *Cyanobacteria* was determined in the ileum at 5 weeks, and a higher abundance of *Proteobacteria* and *Bacteroidetes* was determined at 18 weeks in the ileum at the phylum level. The phylum *Firmicutes* in the cecum contents was highly abundant in the 1-week group, and the phyla *Bacteroidetes* and *Cyanobacteria* were enriched in the 11-week group (*p* ≤ 0.05; Figure 6C). *Enterococcus*, uncultured *Ruminococcaceae*, *Faecalibacterium*, and *Lactococcus* were enriched in the ileum at 1 week (Figure 6B); the higher abundance of *Staphylococcus*, *Brevibacterium*, *Brachybacterium*, *Streptococcus*, *Chloroplast*, *Dietzia*, and *Aerococcus* was determined at 5 weeks; the higher abundance of *Corynebacterium*, *Jeotgalicoccus*, *Facklamia*, and unclassified *Intrasporangiaceae* was determined at 11 weeks; the higher abundance of *Escherichia*-*Shigella* and *Bacteroides* was determined at 18 weeks compared with their levels in pullets of other ages. According to the results obtained from the LEfSe analysis presented in Figure 6D, it is evident that there is a higher abundance of the *Clostridia* vadinBB60 group, *Erysipelatoclostridium*, uncultured *Ruminococcaceae*, the *Ruminococcus* torques group, unclassified *Oscillospiraceae*, *Incertae Sedis Ruminococcaceae*, *Eisenbergiella*, DTU089, *Colidextribacter*, *Oscillibacter*, *Butyricicoccus*, and *Escherichia-Shigella* in the cecum at 1 weeks; a higher abundance of *Lactobacillus*, *Faecalibacterium*, *Alistipes*, and *Blautia* at 5 weeks; a higher abundance of Bacteroides at 11 weeks; a higher abundance of *Lachnoclostridium*, the *Eubacterium coprostanoligenes* group, the *Christensenellaceae* R−7 group, UCG−005, and *Clostridia* UCG−014 compared with their levels in pullets of other ages.

## 4. Discussion

Alpha and beta diversities were employed to assess the similarity or dissimilarity of microbial communities between samples, making them a valuable technique for capturing variations in community composition within the ecosystem. We conducted a diversity analysis using the age of the birds as an exploratory variable to investigate how the bacterial community composition varied in both the ileum and cecum. The NMDS plots for the ileum and cecum regions (except for the weighted UniFrac) evidently illustrated the significant impact of bird age on bacterial beta diversity, as shown in Figure 2 and Figure 3. In a previous research study conducted by Coloe et al. [20], it was reported that the establishment of the cecum microbial community in chickens typically requires a period of around 6 to 7 weeks post-hatching. Subsequently, as the chickens undergo development and growth, it has been observed, as noted by Lu et al. [21], that the microbiome within the ceca undergoes continuous changes and diversification. This dynamic process is integral to the understanding of the avian gut microbiota in the context of poultry development. In their study, Liu et al. [2] demonstrated that the abundance and diversity of the bacterial community in pullets at the age of 12 weeks exceeded that in pullets at earlier time points. These findings imply that the establishment of cecum microbiota in pullets may not be finalized before the 12th week. Consonant with the present study, our empirical findings indicate that the establishment of the microbiota in the pullets was a process extending up to the 11th week. Hume et al. [22] observed that the diversity of the cecum bacterial microbiota in Leghorn chickens increased with age. Bacterial community composition was affected by age, which is similar to the results of Ngunjiri et al. [23] and Wielen et al. [24], who noted that the diversity of the bacterial microbiota in the ileum and cecum of broiler chickens showed a notable increase with age. Thus, the microbiomes of the cecum and ileum constantly change and diversify as a pullet develops and grows. In the current study, our findings indicated that the richness and diversity of the bacterial community in pullets at 18 weeks of age were greater than those at earlier time points.

The results of this study, similar to the findings of Li et al. [25], revealed distinct alterations in the intestinal microbiota of pullets that corresponded to shifts in the growth and developmental stages of the host. Consistent with the results reported by Ballou et al. [26] and Dai et al. [27], our study also noted that, after embryonic microbial succession, the microbiota in the ileum and cecum of pullets at 1 week exhibited relatively low microbial diversity, with *Firmicutes* (respectively, 99.5% and 98.6%) emerging as the predominant phylum. Zhao et al. [28] reported the presence of approximately 37% *Firmicutes* and 10% *Bacteroidetes* in chickens at 8 weeks of age. Nordentoft et al. [29] conducted a microbiota characterization of hens at 18 weeks of age, revealing that the predominant microbiota consisted of representatives from the *Firmicutes* and *Bacteroidetes* phyla. In addition, there were smaller populations of *Proteobacteria*, *Actinobacteria*, and *Fusobacteria*. Meanwhile, *Firmicutes* and *Bacteroidetes* remained the two most abundant phyla in the cecum of pullets for 12 weeks [2] and 16 weeks [11], which is consistent with earlier studies. Through linear discriminant analysis, enriched *Firmicutes* were identified as biomarkers associated with the acceleration of intestinal development [27]. The *Bacteroidetes* abundance exhibited a resurgence, concomitant with an increase in the relative abundance of *Lactobacillus* and a rise in the diversity of the microbial community. These modifications can lead to a decrease in the pH of the intestinal lumen, hindering the colonization of pathogenic bacteria while simultaneously improving nutrient utilization. As a result, these changes contribute to the heightened egg production observed during the laying period [9,30]. Various microorganisms possess unique metabolic functions, suggesting that the gut microbiota may co-evolve with their host animals and subsequently affect the various physiological functions of these hosts [2]. *Firmicutes*, the dominant phylum present in both the cecum and ileum across all age groups, are responsible for fermenting non-digestible dietary carbohydrates into short-chain fatty acids (SCFAs), including butyric acid. These SCFAs serve as an energy source and contribute to the reinforcement of the intestinal barrier [31]. An elevation in *Firmicutes* levels is indicative of a concurrent rise in the production of butyric acid, a byproduct of microbial fermentation. Butyric acid has the capacity to bind to G protein-coupled receptor 43, which is present on dendritic cells, triggering the activation of the Wnt signaling pathway within bone marrow stromal cells [31]. This activation results in an increased production of TGF-β by Tregs, subsequently promoting their proliferation and differentiation into osteoblasts. These intricate interactions contribute to bone development and health [32]. The pivotal phase for accelerated bone development in layer chickens occurs during the period of 6 to 11 weeks post-hatching. Hence, the intermittent rise in the population of butyric acid-producing bacteria appears to align with the physiological requirements associated with the rapid bone development observed during the pullet phase. *Lachnospiraceae* and *Ruminococcaceae* were additionally identified as having a strong correlation with feed efficiency in poultry, as highlighted in the study by Singh et al. [33]. *Bacteroides* (5.39−8.49%) and *Alistipes* (4.46−7.96%) comprised the predominant bacterial composition in the cecum contents of pullets at 5, 11, and 18 weeks of age. *Bacteroides* are involved in the degradation of the isoflavone genistein [34]. Gauffin et al. [35] suggested that genistein ameliorates metabolic and immunological issues in mice with diet-induced obesity. These findings imply that a higher abundance of *Bacteroides* may have a positive effect on gut health. Furthermore, *Alistipes* was distinguished via its significant expression of xylose isomerase and glutamate decarboxylase, enabling the conversion of glutamate into another essential short-chain fatty acid known as γ-aminobutyric acid [36]. In our study, *Lactobacillus* (12.17−67.01%) constituted one of the predominant genera in the ileum across different age groups. Ndotono et al. [37] and Ngunjiri et al. [23] found that *Lactobacillus* species belonging to the family *Lactobacillaceae* were the predominant bacteria in the chicken ileum in their respective studies. This bacterial family comprises beneficial commensals that have been extensively studied in both the human and animal food and medical industries [18]. In the context of health, these lactic acid-producing bacteria have been extensively reported to confer various health benefits. Among these advantages is the ability to stimulate immune responses, exhibit anticancer activity, contribute to the prevention and treatment of inflammatory diseases, alleviate lactose intolerance, demonstrate effective antimicrobial properties against resistant pathogens, and address respiratory viral infections [38,39] In addition, *Lactobacillus* species can produce lactic and acetic acids, leading to a decreased gut pH [18]. This mechanism facilitates direct competitive interactions with potential pathogens, allowing them to outcompete these pathogens for vital nutrients [39]. Consequently, *Lactobacillus* species effectively inhibit the colonization of specific significant pathogens, contributing to gut health. In the context of our study, it appears that *Lactobacillus* and other dominant bacteria may play pivotal roles in addressing the distinctive physiological developmental requirements of pullets during this phase when preparing for egg production.

Opportunistic infections are caused by microorganisms that do not normally cause disease. However, they become pathogenic when the chicken immune system is weakened and unable to fight infection. A compromised immune system provides an opportunity for pathogens (bacteria, viruses, fungi, or protozoa) to infect the individual [40]. Common opportunistic pathogens include *Staphylococcus*, *Streptococcus*, *Enterococcus*, *Ruminococcaceae*, *Alistipes*, and *Escherichia-Shigella* [41]. Our study findings reveal that *Streptococcus* (1.38−3.92%) and *Staphylococcus* (22.65−29.53%) populations in the ileum constitute a significant portion of intestinal microorganisms at 5 and 11 weeks. Joat et al. [42] reported that the abundance of the Streptococcus genus was greater among pullets raised on the floor compared to that in those raised in cages. Encouraging the growth of intestinal microorganisms via the use of probiotics, prebiotics, and related substances presents an opportunity to restore balance within the gastrointestinal microbial community, thereby reducing the risk of diseases caused by opportunistic bacteria [43].

## 5. Conclusions

This study examined alterations in the gut microbiome of pullets throughout the rearing period in a floor environment. The four growth stages were found to play a significant role in influencing the alterations observed in the gut microbiota of pullets. The study underscores the importance of effectively controlling pathogens and beneficial bacteria during the initial growth stage (0−11 weeks) for floor-raised pullets. The inferred functions of the gut microbiota suggest potential roles of the cecum and ileum microbiota in metabolic regulation and developmental processes of pullets at the rearing stages, which may have implications for the utilization of these findings in the context of probiotic supplementation. Furthermore, the data generated in this study can serve as a reference for comparative investigations into other hens.

## Figures and Tables

**Table 1:** The alpha diversity of the ileum microbiome during different stages of pullets for the first 18 weeks.

Week	Chao1	Shannon Entropy	Simpson	Faith pd
1	65.7 ^b^	3.44	0.81	3.52 ^b^
5	60.0 ^b^	4.28	0.92	3.80 ^b^
11	138.9 ^ab^	4.26	0.87	6.13 ^b^
18	191.7 ^a^	4.15	0.75	18.30 ^a^
SEM	28.63	0.561	0.053	2.232
*p* value	0.0139	0.6847	0.1825	0.0006

SEM, standard error of means. ^a,b^ Means in the same column with different superscripts are significantly different (*p* ≤ 0.05).

**Table 2:** The alpha diversity of the cecum microbiome during different stages of pullets for the first 18 weeks.

Week	Chao1	Shannon Entropy	Simpson	Faith pd
1	155.1 ^b^	5.72 ^c^	0.96 ^b^	8.28 ^d^
5	286.9 ^b^	6.36 ^b^	0.97 ^b^	14.94 ^c^
11	667.5 ^a^	7.56 ^a^	0.99 ^a^	28.46 ^a^
18	569.8 ^a^	7.52 ^a^	0.99 ^a^	23.94 ^b^
SEM	45.40	0.135	0.003	1.324
*p* value	<0.0001	<0.0001	<0.0001	<0.0001

SEM, standard error of means. ^a–d^ Means in the same column with different superscripts are significantly different (*p* ≤ 0.05).

## Data Availability

Data are contained within the article.
